# Dough Performance and Quality Evaluation of Cookies Prepared from Flour Blends Containing Cactus (*Opuntia ficus-indica*) and Acacia (*Acacia seyal*) Gums

**DOI:** 10.3390/molecules27217217

**Published:** 2022-10-25

**Authors:** Shahzad Hussain, Mohamed Saleh Alamri, Abdellatif A. Mohamed, Mohamed Abdrabo Ibraheem, Akram A. Abdo Qasem, Ghalia Shamlan, Ibrahim A. Ababtain

**Affiliations:** Department of Food Science and Nutrition, King Saud University, Riyadh 1145, Saudi Arabia

**Keywords:** cactus, acacia, texture, cookies, sensory

## Abstract

Acacia (AG) and cactus gums (CG) were mixed into wheat flour at the 3% and 6% levels. The flour blends were tested for their pasting, dough development, and extensibility behaviors. The blends were used to make cookies, which were then evaluated for their physical, textural, and sensory qualities. Both types of gum reduced the setback viscosities, water absorption, and farinograph quality numbers while increasing the water retention capacity, dough development time, and extensibility. The thickness and diameter of the cookies decreased in the presence of the cactus gum, while the acacia gum resulted in greater thickness and diameter. The addition of more gums increased the hardness of the cookies while decreasing their fracturability. All the cookie types were acceptable for all the sensory attributes studied. When compared to the control, the panelists preferred the color of the cookies with a higher level of gum. Overall, the presence of gums in the formulation resulted in the development of cookies with improved technological and sensory attributes. Likewise, the cookies with higher levels of gum can deliver 6% more soluble fiber without compromising their overall acceptability.

## 1. Introduction

Cookies are the most popular bakery items in many parts of the world, and they are enjoyed by people of all ages, including children, teenagers, and the elderly [1]. Cookies are thought to have good nutritional values; they have a long shelf life, are easy to eat, and come in a variety of ingredients, textures, and flavors. Cookies are traditionally made with three basic ingredients: flour, sugar, and fat. They are also made for special dietary needs, using ingredients that are high in dietary fibers, antioxidants, and prebiotics; they have low or no gluten, and they have other health benefits [2,3]. Cookie components may behave differently when used together. When the various ingredients in the dough are added, the processing parameters must also be adjusted. The texture and sensory attributes of the cookies are the key factors being controlled in order to provide the best quality product to the consumers. The nature of the ingredients and the processing parameters influence the dough properties and cookie quality. Many researchers have studied the use of various ingredients, such as beta-glucan, cordia and ziziphus gums, wheat malt, sesame peel flour, guar gum, polydextrose, and so on, in varying concentrations [4,5,6,7,8].

Hydrocolloids are polysaccharides composed of simple sugar building units 6–8. These polysaccharides are widely used in the food industry as gelling, stabilizing, thickening, and suspending agents. Each of them contains hydrophilic molecules, which, when combined with water, result in the formation of viscous solutions [9,10]. Cactus mucilage is a slimy substance extracted from cactus cladodes; it has a high molecular weight of approximately 3.67 × 10^6^ g/mol and is well known for its hydrophilic nature [11,12,13]. Cactus gum is made up of acidic fractions that include arabinose, galactose, rhamnose, xylose, and galacturonic acid, as well as neutral fractions that include glucans and glycoproteins [14]. It can be used as an ingredient to improve food texture and to alter the pasting and thermal behavior of various starches. Cactus mucilage is used as an additive hydrocolloid to improve the baking performance of breads and cakes and to improve the pasting and thermal properties of starches [15,16]. Gum Arabic, also known as acacia gum, is derived from various acacia plant species, most notably A. Seyal and A. Sanegal of the Leguminosae family. It has long been used as an additive in a variety of food products, as either a foaming agent, an emulsifying agent, or an encapsulating material [17]. It is considered to be a non-toxic, edible, tasteless, and odorless natural compound mainly comprising high molecular weight polysaccharides (250,000) and their potassium, calcium, and magnesium salts, which on hydrolysis yield glucuronic acid, rhamnose, galactose and arabinose. Galactose (39–42%), arabinose (24–27%), glucuronic acid (15–16%), and rhamnose (12–16%) make up the bulk of the chemical composition of the acai gum [18]. According to Maier et al. [19], it can be added to bakery products as well as flour to increase the shelf life and retain moisture. Because of its high polymeric nature and the interaction between polymeric chains when dispersed, it can also be used to change the viscosity of the system [15,20]. The current study was carried out to investigate the effect of locally collected acacia (AG) and cactus (CG) gums on dough performance and cookie quality attributes and sensory properties.

## 2. Results and Discussion

### 2.1. Pasting Properties of Flour Gum Blends

The pasting properties of the wheat flour blended with cactus gum (3, 6%) and acacia gum (3, 6%) were evaluated using the Rapid Visco-Analyzer (RVA), and the data are presented in Table 1. The RVA profiles of the blends present the peak viscosity (PV), peak time, breakdown (BD) and setback (SB), final viscosity (FV), and pasting temperature. Regarding the PV, the highest viscosity value (1939 cP) was noticed for the wheat flour with the addition of 6% cactus gum, which surpassed the value of the control wheat flour (1888 cP). PV is an indicator of the maximum swelling of the starch granules in the wheat flour with the imbibition of water under applied shear and heat. However, when either the 3% or the 6% acacia gum was present, the PV dropped significantly and drastically. The lowest PV was seen in the wheat flour blends with 6% acacia gum, where the PV dropped by almost 41%. When comparing both of the gums, the acacia gum provided stronger reduction in the PV than the cactus gum, indicating that the acacia gum strongly coated the starch granules and hindered their swelling and integrated the granular structure [21]. Hence, it indicates that the type of gum and its concentration manipulates the level of the extent of the interaction with the wheat flour and modulates the PV of the blends. Similarly, in a previous report, the reduction in the PV of the wheat flour was noticed with the addition of ziziphus gum [22].

In contrast to the PV, the FV of the control wheat flour was the highest among all the samples. The reduction in FV was higher for the samples with a lower concentration of gums (i.e., 3%). Interestingly, the flour blends with 6% gum acacia presented the same FV as of the 6% cactus gum with a value of 2028 cP. However, the lowest FV (1592 cP) was noticed for the blend of wheat flour with 3% acacia gum, leading to a reduction of 22% in the viscosity of the paste. In the RVA profile, the FV of the blends is estimated at the end of the holding period at 50° C, and it is mainly a result of the alignment and rearrangement of the percolated amylose and the short chain amylopectin from the starch granules under heat. This reformation and aggregation of the leached fractions of the starch finally results in the formation of wheat flour gels [23]. Alamri et al. [24] suggested that the reduction in FV might be correlated with the lower amount of the total starch percentage in the blend due to the replacement with the gums. In line with our study, the reduction in the FV is also in line with the literature report in which ziziphus gum and Arabic gum lowered the viscosity of the wheat flour and starches [6,22,25]. 

The BD viscosity value is an indirect estimator of the stability of the flour paste under shear and heat, and in the RVA profile, it sets off after the PV. The lower the BD, the higher the stability of the blend and the better the resistance against the applied temperature and shear. In line with the PV, the BD viscosities of the samples were noticed; the highest value (749 cP) was noticed in the wheat flour blend with 6% cactus gum added, and the lowest (445 cP) was in the sample with 6% acacia gum. In other words, the most stable wheat flour gel was seen when there was 6% acacia gum in the blend. In particular, the gums with the lower concentrations in the blends imparted better resistance and stability and resulted in lowered BD. Apart from the 6% cactus gum addition, all the other levels of gums (acacia and cactus) better coated the starch granules and controlled their imbibition and rupturing and thus maintained the paste structure. In line with the current data, the lower BD was also noticed when the ziziphus and cordia gums were added to the wheat flour, improving the tolerance of the blend system [22]. 

In the case of the SB, the highest value of 902 cP was noticed for the control wheat flour without the presence of any gum. However, a lowering of the SB was noticed with the addition of both types of gums at both concentrations (i.e., 3 and 6%). In the RVA profile, the SB is marked as the viscosity difference between the FV and the BD, and it is achieved when the temperature of the paste is reduced from 95 to 50 °C and the leached amylose chains start the aggregation and recrystallization. Interestingly, the higher SB was noticed for the blends with 3% cactus gum while the lowest setback was noticed for the blends with 6% acacia gum. It is noteworthy that the acacia better controlled the recrystallization of the starch than the cactus. Thus, the addition of acacia hindered the amylose–amylose interaction by plasticizing and interfering in the network of the gel structure. Hence, to obtain a softer gel a higher concentration of the acacia gum would be better and would provide a more pliable texture. In the literature, a lowering of the SB is also reported for the wheat flour gel and starches when the acacia and the cordia gum were admixed into the blend [22,25]. 

In the RVA profile, PT corresponds to the temperature at which an initial rise in the viscosity of the wheat flour blends is noticed under the applied shear. The PT shows the magnitude of heat energy which is required to gelatinize the starch. The highest PT of 84.9 °C was observed for the blends when acacia gum was present at both the levels of addition. On the other hand, the lowest PT was noticed for the samples with the addition of cactus gum. This specifies that the sample with acacia gum possesses a higher gelatinization temperature and will also need a higher cooking temperature compared to that of the wheat flour. This higher PT for the wheat flour samples with acacia indicates a slower and delayed imbibition and swelling of the starch granules due to the coating of the granules [21]. Thus, it indicates that the acacia gum improves the thermal stability of the wheat flour system. Interestingly, the increase in the concentration of both the gums from 3 to 6% did not increase the PT of the wheat flour, indicating a saturation effect where the interaction sites of the starch granules were fully occupied by the gum at a 3% level of addition. Hamdani et al. [26] have also demonstrated a rise in the PT when chickpea and rice flour was added with guar gum and locust bean gums at 1%. Conversely, a reduced PT of the wheat flour was observed when native and acetylated cordia gum was added to the wheat flour [22,23]. In the case of peak time, the longest time was noticed for the blends with cactus gum added, whereas wheat flour with acacia gum depicted the fastest peak time. In line with the current report, Hamdani, Wani, and Bhat [26] also observed a shorter peak time for the blends of chickpea with locust bean and guar gum.

### 2.2. Dough Mixing Properties of Flour Gum Blends

The dough mixing properties, such as water absorption (WA), dough development time (DDT), the mixing tolerance index (MTI), dough stability, and the degree of softening of the wheat flour blends with the gums, were estimated and data are reported in Table 2. Dough is developed when the major proteins in wheat flour, such as gliadin and glutenin, are hydrated and form a gluten network where the starch and minor fractions of flour fill up the protein network. The dough properties are critical in designing baked products of the desired organoleptic characteristics. 

The WA of the flour to develop dough indicates the water amount required to obtain a dough consistency reaching to 500 FU. It is mainly defined by the quality and concentration of the gluten in the wheat flour blend system. In addition, the presence of non-protein fractions also manipulate the WA and the optimum dough development. In the current study, the highest WA of 61.47% was noticed for the control wheat flour. However, the addition of gums in the flour reduced its water absorption and the lowest WA (56.0%) was noticed for the sample with 6% acacia added. The reduced WA of the blends could be due to the dilution of the glutenin fraction of the flour in the presence of the gums, which resulted in a poor formation of the dough network [27]. Chen et al. [28] also alluded to the fact that the glutenin plays a more significant role than the gliadin in the WA and retention during baking. The lowering of the WA was more for the samples with the acacia gum compared to those with the cactus gum. However, for a given gum, the change in the concentration of the gum did not modify the WA of the wheat flour blend. The current result of the higher WA of wheat flour than the other blends could be related to the higher gluten protein and lower fiber content. A reduction in the WA was also reported when soluble hydrocolloids from *Cordia myxa* (L.) were incorporated into the wheat flour [22]. 

The DDT was the lowest (1.6 min) for the control wheat flour compared to the other formulations with both gums. DDT is the time to reach the maximum consistency during dough preparation. Compared to the control, a significant rise in the DDT was noticed with the addition of gums to the blend. However, a four-fold upshift in the DDT was noticed for the wheat blends prepared with 6% acacia gum. This delayed DDT of the dough could be attributed to the hindered hydration of the protein fractions in the developing dough and the water competition of the acacia gums with the glutenin and gliadin. However, for both the gums, a dose-dependent rise in the DDT was noticed when the gum concentration was increased from 3 to 6%. In line with the current study, the rise in the DDT of the dough occurred by incorporating the water-soluble resistant dextrins at higher concentration (10%) [29]. Liu et al. [30] also reported a rise in the DDT when xanthan gum was added to the gluten-free flour blends. 

Dough stability imitates the mechanical strength of the dough and the retention of the consistency under the shear over a given period. The highest stability was noticed for the sample with 3% acacia gum added, followed by the control wheat flour, which was like the dough blend with 6% acacia gum; however, the lowest stability was shown by the sample with the 6% cactus gum addition. It shows that the most durable and stable dough network was obtained by the acacia gum addition, which surpassed the control wheat flour dough stability. These stability results were also supported by the lowest PV being that of the blends with the acacia gum in this study. However, in comparing the gums, the blends with 3% and 6% acacia depicted almost double the stability compared to that of their counterparts containing 3 and 6% cactus gum. Hence, the acacia gum presence strengthened the dough structure and enhanced the durability under shear. Azeem et al. [31] reported a decline in the stability of the dough upon the addition of various hydrocolloids into sweet potato–wheat flour blends.

The MTI of the dough is the estimation of the degree of tolerance and resistance before the weakening the dough network. In the mixogram, it is represented by the difference in the height of the maximum consistency and that after 5 min of mixing and is given in farinograph units (FU). The MTI ranged between 35.3 FU and 117.3 FU, where the highest was noticed for the blend with 6% cactus gum and the lowest MTI was shown by the control wheat flour. The lower MTI of dough is considered good for developing fermented bakery products. Thus, the control wheat flour depicted the best resistance to mixing force while the cactus gum samples remained the least durable. Among the gums, the acacia positively contributed to the stability of the dough. Interestingly, the softening pattern was nearly same with the MTI in this study, corroborating the poor resistance of the doughs blended with gums. Interestingly, the cactus gum dough provided almost double the MTI of the sample with the acacia gum. Overall, both the gums could not preserve the dough durability compared to the control as they presented higher MTI values. Generally, dough with a higher MTI provides poor dough handling properties and imparts inferior mechanical strength. In line with the current results, the addition of ziziphus gum worsened the dough strength and provided a higher MTI, as reported by Alamri, Mohamed, Hussain, Ibraheem, Qasem, Shamlan, Hakeem, and Ababtain [22].

Dough softening is an indirect estimator of the strength and durability. Thus, the dough blend with the highest stability should depict an inverse relationship with the softening values. In the results, the most stable and durable dough of the wheat flour blended with acacia gum presented the lowest softening, at 75.0 FU, which was less than the control wheat flour. This lower degree of softening of the wheat flour blends with the acacia augmented the data for stability in this study. The highest softening (152.6 FU) was noticed for the sample with 6% cactus gum, followed by the blends with 3% acacia (146.0 FU). Interestingly, the softening of the dough with 6% cactus was roughly double that of the dough with the 6% acacia replacement. Overall, acacia performed better and controlled the dough softening by interacting with the gluten proteins by hydrogen bonding. Generally, the MTI and softening are positively correlated, where an increased MTI depicts an increase in the softening of the dough [32]. 

The quality number of the dough in the mixogram is the distance (in mm) between the point of water addition and the point where the central line lowers to 20 FU as a function of time. This is an indicator of the dough’s stability and resistance against kneading. The highest quality number was noticed for the control wheat flour, with a value of 61.2 mm, followed by the dough with acacia gum and then by the dough with the cactus gum. The least stable dough was the sample containing 6% cactus gum. This reduction in the quality number could be due to the gluten–gum interactions occurring instead of the gluten–gluten connections that might be present in the control dough [22].

### 2.3. Dough Extensibility Properties of Flour Gum Blends

**Figure 1:**
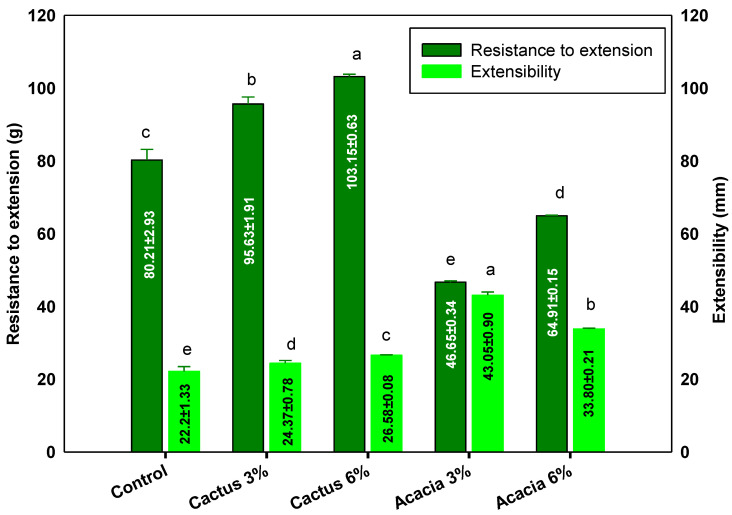
Dough extensibility properties of flour gum blends. Values followed by different letters in same color bars are significantly different at *p* < 0.05.

Dough extensibility (mm) is the dough stretching extent before the breakage of the network structure. It gives a good measure of the dough strength and is an estimator of the dough consistency and of the quality of the finished baked product. Dough extensibility and elasticity are dough balancing acts which start from the dough mixing, followed by the development of the gluten cohesive matrix, which thus imparts the elastic and extensible properties to the dough. The Figure 1 graph indicates that the lowest extensibility (22.2 mm) was presented by the control wheat flour dough without any gum. On the other hand, the highest extensibility (43.05 mm), which was double that of the control, was seen for the dough blend with 3% acacia gum. This shows that the control wheat flour developed a cohesive viscoelastic gluten network through intra- and intermolecular interactions, which resulted in a relatively less stretchable dough, whereas the presence of gums modified the interactions and resulted in more pliable and relatively extensible network, particularly with the acacia gum at 3 and 6%. 

The dough matrix is a combination of two phases: a continuous phase of high molecular weight glutenin held by disulfide linkages, which impart the elastic nature, and a discontinuous phase developed by low molecular weight gliadin, which imparts the viscose nature. The added gums, due to their lower molecular weight compared to glutenin, align themselves with the discontinuous phase of the dough and contribute to balancing the elasticity and extensibility [33]. Doughs with lesser extensibility are considered better for cookie preparation, while more elastic dough is favorable for bread preparation, where a higher gas volume needs to be incorporated and retained by the gluten network [34,35]. In line with the current results, Mohamed, Hussain, Alamri, Ibraheem, Qasem, and Ababtain [33] reported improved dough extensibility with the addition of ziziphus gum. In contrast to our result, a higher extensibility of the dough was imparted by the added xanthan gum in the wheat flour [36]. 

Resistance to extension is an important factor for dough quality, and it estimates the dough strength and gas-holding capacity. The addition of cactus gum at a higher percentage resulted in the least-elastic dough network, with the highest value of 103.15 g. Conversely, the presence of acacia at either 3% or 6% in the wheat flour impaired the resistance to extension of the developed dough. The cactus gum provided two-fold higher resistance to extension, suggesting that the increased hardness of the dough and more energy would be needed for the desired product preparation. Thus, with the addition of both gums, the extensibility was primarily presented by the gummy nature of the prepared dough, while the resistance to extension of the dough was reflected by the dough hardness with the cactus gum. The most desirable dough qualities are rendered by a mix of extensibility and sufficient resistance [37]. In a previous report, the addition of 5% cordia gum to the wheat dough resulted in a less elastic dough with the highest resistance to extension [33]. Hence, based on the extensibility data, acacia gum is more favorable in the production of cookie dough compared to the cactus gum.

### 2.4. Solvent Retention Capacity (SRC) of Flour Gum Blends

**Figure 2:**
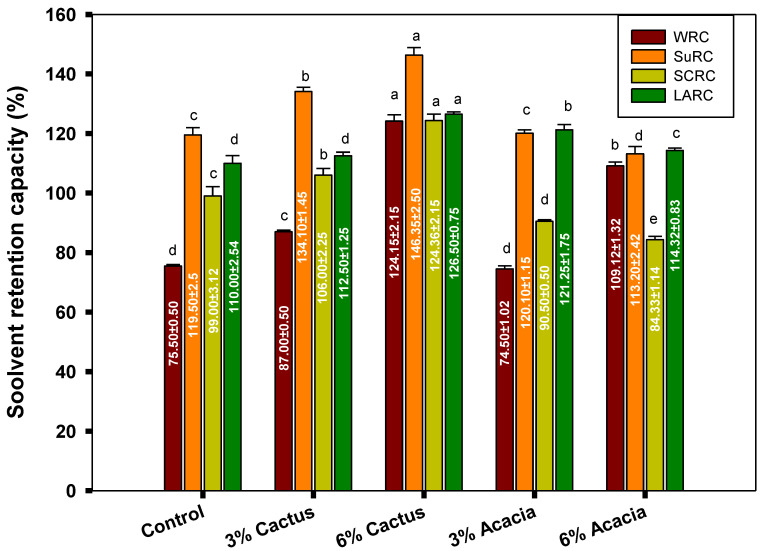
Solvent retention capacity of flour gum blends. WRC = water retention capacity; SuSRC = sucrose retention capacity; SCRC = sodium carbonate retention capacity; LARC = lactic acid retention capacity. Values followed by different letters in same color bars are significantly different at *p* < 0.05.

The solvent retention capacity of the wheat flour and blends with acacia and cactus gums is presented in Figure 2. The SRC provides a fruitful profile of the flour blends’ qualities and possible functionality in order to estimate their baking performance. The SRC quantifies the extent of the swelling of the polymer networks of flour or blends by retaining the given solvent and relating it with the functionality related to the specific fractions found in the blends. Thus, based on the specific functionality, the flour or blends could be opted for in a particular baking application. Here, in this study, four types of solvent retentions were estimated, namely water (WRC), sodium carbonate (SCRC), sucrose (SuRC), and lactic acid (LARC). WRC is related to the total functional components of the flour blends, such as starch, glutenin, gliadins, arabinoxylans, etc. [38,39].

The WRC for the blends with 6% cactus gum remained the highest, with a value of 124.1%, whereas the lowest WRC (75%) was seen for the control wheat flour with no added gums. For a given gum, i.e., acacia or cactus, a higher concentration of gum resulted in higher WRC for the blends, depicting a dose-response effect. Thus, the presence of the gums imparted higher WRC to the blends, which could be attributed to the high molecular weight of the gums and their stronger hydrophilic nature. In other words, even the blends have diluted fractions of proteins, starch, and other wheat components; yet, the presence of the gums augmented the WRC by holding higher total weight of water. In line with these results, a greater level of addition of ziziphus and cordia gum in the wheat flour augmented the WRC [33]. The liquid retention of the flour and blends greatly influences the ingredient functionality, shelf stability, and product yield. The quality of baked goods is strongly manipulated by the presence of water, as it is by the starch gelatinization and retrogradation, protein unfolding, and yeast activation in the dough mixing and baking [40]. 

As with the WRC, the highest SuRC was observed for the blends with the addition of 6% cactus gum, while the lowest was depicted by blend with the addition of 6% acacia gum. The SuRC is an indicator of the pentosan concentration and functionality in the blend [40]. The SuRC is special in the sense that it imitates the functional environment in the cookies or in the dough for high-sugar crackers. In line with higher SuRC in this study, the addition of cordia gum also improved the SuRC of the cookies made from wheat flour [33].

The SCRC estimation of the flour relates to the existence of the level of damaged starch [39]. The SCRC followed a somewhat similar pattern to that of SuRC, where the blends with 6% cactus gum depicted the highest holding of sodium carbonate, whereas the lowest percentage of retention was observed for the blends with 6% acacia. Thus, it shows that the flour blend with 6% cactus gum mimics the presence of a higher amount of damaged starch, which has the ability to hold a higher number of solvent molecules by establishing hydrogen bonding [41]. A better retention of sodium carbonate in the blends suggests that protonated sodium carbonate or baking soda will be better dispersed in the cookie dough and will provide a good leavening effect. A higher SCRC of the wheat flour was also seen when the blends were made with 5% ziziphus and cordia gum [33]. The SCRC pattern is directly correlated with the flour performance during baking for various end-use applications. 

Similarly, the highest amount of LARC (126.5%) was noticed for the blends with 6% cactus gum and the lowest was noticed for the control wheat flour (110%). Compared to the cactus gum blends, the acacia blends stood second in the LARC with a value of 121.2%. The LARC of the blend provides the estimation of the glutenin network formation and the strength of the gluten. Mohamed, Hussain, Alamri, Ibraheem, Qasem, and Ababtain [33] reported a higher LARC of 174% for wheat flour blends when added with 5% of ziziphus gum, whereas the addition of cordia gum drastically downregulated the LARC to a value of 97.5%, which is lower than all the blends in this study. Thus, the gum type and level of addition could manipulate the blend properties and would be suitable for certain baking applications due to their difference in the WRC, SuRC, SCRC, and LARC. For instance, a flour blend with 51% WRC, 89% SuRC, 87% LARC, and 64% SCRC may be suitable for cookie manufacturing; however, 57%, 96%, 100%, and 72% of WRC, SuSRC, LARC, and SCRC, respectively, would be effective for dough and sponge development [42].

In the current study, the suitability of the flour for cookie preparation could be seen with the values of the SuRC (119.5%), LARC (110%), SCRC (99%), and WRC (75%); the higher value of the LARC of the flour achieves the criteria of soft wheat, which is suitable for crackers, while the high SuRC imitates the functional environment of cookies. Thus, the source of the gum and its concentration have significant roles in changing the retentions of all four solvents. Therefore, the differences in the gum effects on the wheat flour quality could easily be authenticated by the SRC, which could idealize the best end-use functionality of the wheat flour [43].

### 2.5. Textural and Physical Properties of the Cookies

The cookie texture, i.e., the hardness and the fracturability, was estimated using a texture analyzer (Table 3). Hardness (g) is the peak force which is applied to break the cookies. The cookies prepared from the wheat flour blended with 6% cactus gum showed the highest hardness (5110 g), followed by the cookies with 3% cactus gum. However, an almost 50% reduction in hardness was noticed with the addition of the 3% cactus gum compared to the 6% gum. The softest cookies were the ones baked with the control wheat flour without the addition of any gum; they had the lowest textural hardness value of 2408 g. The low hardness of the control cookies could be due to the uniform network development of the proteins with a uniform pore size, due to moisture evaporation during the baking process. However, the higher hardness of the cookies in the presence of the cactus gum could be correlated with the dilution of the proteins and the formation of the aggregation of the gum in the presence of moisture, which resulted in an irregular distribution of pores and hence imparted an overall higher hardness. Increased hardness was also reported by Lou et al. [44] when grape pomace powder was supplemented in the wheat flour. Conversely, Hamdani, Wani, and Bhat [26] reported on lowering hardness with the addition of locust bean and guar gum in the cookies.

The fracturability of cookies is recorded as the distance travelled by the probe to the point of fracture. The fracturability of the cookies varied between 0.37 mm and 0.65 mm, where the lowest fracturability was noticed for the control wheat flour cookies and the highest fracturability was shown by the cookies with the addition of 6% acacia gum. The fracturability has a strong effect on the crispiness of the cookies. The increased fracturability of the cookies with the added gums is considered to be due to the emulsification properties of the gums. In a study, the addition of guar gum at increasing concentrations enhanced the fracturability of the biscuits [45]. Similarly, fiber- and protein-enriched biscuits were prepared with a sorghum–wheat composite flour, and a higher fracturability was noticed due to the addition of spirulina (Spirulina platensis) powder [35].

The dimensions of the cookies, such as their thickness and diameter, effect their organoleptic properties. The highest diameter and thickness (longitudinal and vertical expansion) were noticed for the cookies prepared from the blend with the addition of 6% acacia, while the lowest diameter and thickness were presented by the cookies with 6% cactus gum in the formulation. The water-holding capacity of the dough is a strongly influential factor which could contribute to higher dimensional expansion [26]. Similarly, the obviously higher water-holding capacity of the formulation with 6% acacia gum clearly led to the cookies having the highest diameter and thickness. In addition to the water-holding capacity, under the given baking conditions, the ingredients in the formulation of the cookies also contributed to the final dimension of cookies. The spread ratio is a physical attribute which estimates the extent of the rising ability of the cookies; it is the ratio of the thickness to the diameter of the product. A higher spread ratio is an indicator of the better quality and acceptability of the product [46]. The highest spread ratio of 6.18 was noticed for the cookies with 6% cactus gum during baking, while the lowest was noticed for the cookies manufactured by the formulation with 6% acacia gum. However, the control wheat flour cookies presented the second lowest spread ratio.

The moisture holding of the formulation had a strong effect on the spread ratio due to the presence of the gums. The formulation with the higher water-holding capacity would dissolve more sugar, and hence, the higher sugar would produce extra water and would lower the viscosity of the dough and raise the initial dough expansion and flowability during baking. Other than that, the release of gases from the leavening agents, the dough consistency, the spread onset time, and the cookies’ setting time are also key determinants for the control of the spread ratio [47]. It is assumed that the viscosity of the gum solution reduces with the rise in the temperature [48]; thus, during baking when the temperature is high, the dip in viscosity and the increased gravitational flow of the cookie dough with added gums might elevate the spread ratio compared to that of the control wheat flour dough. Hamdani, Wani, and Bhat [26] also reported the rise in the spread ratio of biscuits with an increasing concentration of added locust bean gums. In contrast to our results, with the addition of resistant starch III and IV to the cookies, a reduction in the spread ratio was noticed [49].

### 2.6. Color Parameters of the Cookies

**Figure 3:**
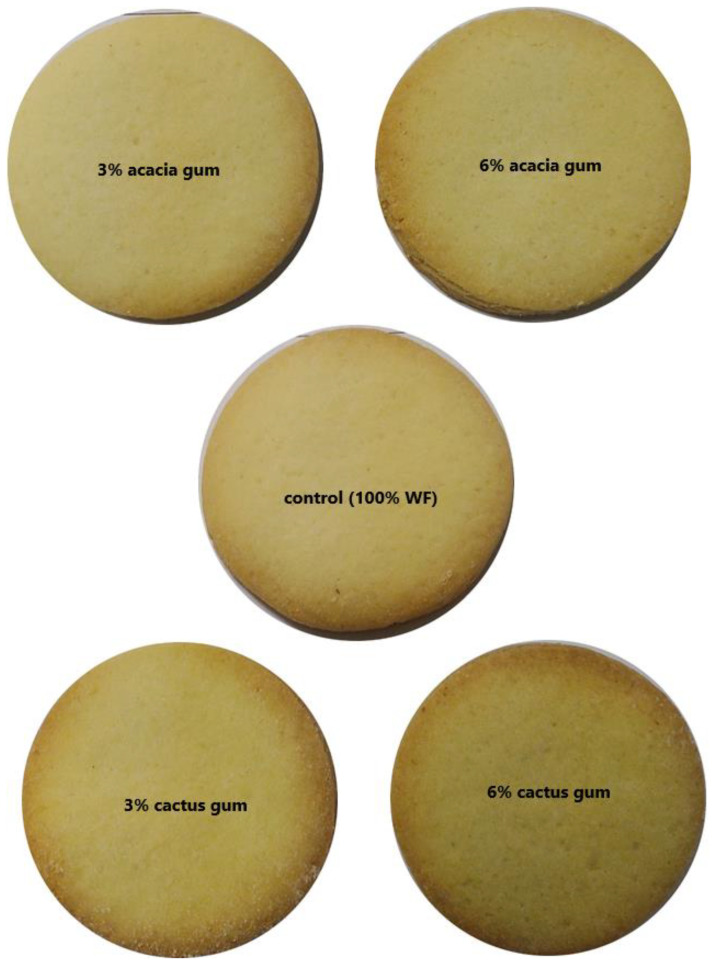
Pictures of cookies prepared from flour gum.

The color components (L* (lightness), a* (greenness), b* (yellowness)) of the control wheat flour cookies and the cookies with the wheat flour and gum blend were estimated and are presented in Table 4. The highest L* value was noticed for the control cookies and for the cookies with 3% acacia gum. On the other hand, the lowest L* value (76.71) was observed for the cookies manufactured from the flour blended with 6% cactus gum. However, compared to the 3% gum, the addition of 6% gum to the wheat flour cookies significantly lowered the L* value. These lower L* values are justified by the higher a* values for the cookies with higher concentrations of both of the gums. The increased a* and reduced L* might be due to the formation of color pigments as a result of non-enzymatic browning during baking. On the a* scale, the lowest value was noticed for the cookies prepared without any gum. In terms of b*, the control sample with no added gums showed the highest value of 28.06, indicating the highest yellowish tinge, whereas the lowest were found for the cookies containing a higher percentage (6%) of both of the gums. Thus, the darkest sample was the one prepared with 6% cactus gum, as is also shown in Figure 3. Mohamed, Alamri, Hussain, Ibraheem, Qasem, Shamlan, and Ababtain [6] reported the reduction in L* for the cookies with the added cordia and ziziphus gums, where the darkest sample was the one with the addition of 2% ziziphus gum.

### 2.7. Sensory Evaluation of the Cookies

**Figure 4:**
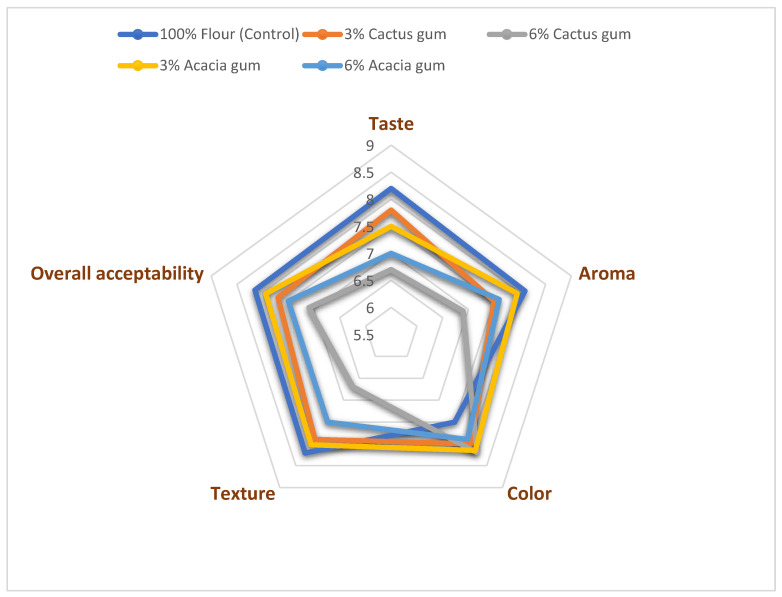
Sensory scores assigned to cookies.

The hedonic average rating of the sensory characteristics of the cookies, such as color, taste, aroma, and texture, and their impact on their overall acceptability is presented in Figure 4. The color is the first and foremost appealing factor of the bakery products, which is attributed to the spectral distribution of light emerging from the surface of the cookies. In bakery products, the development of color is mainly attributed to non-enzymatic browning, such as that via the Maillard reaction. The cookies prepared with wheat flour blended with 6% cactus gum were ranked the best by the panelists. However, a similar ranking was obtained by the cookies manufactured using the blend with 3% acacia gum. Among all the samples, the cookies baked from wheat flour received the minimum likelihood, with a score of 6.5. The better preference for the cookies with the gum could be attributed to the presence of some protein and reducing sugar fractions in the gums and to the better water-holding capacity of the gums. In addition, once heat was applied the formation of the Maillard products would be higher, which might have contributed to the brown color formation in the brown color, which was appealing to the panelists. The lowest L* value of the cookies with 6% cactus gum also supported this trend of color development. 

Taste and aroma combine to develop the flavor and both are associated with small molecular aromatic compounds which are perceived by the sensory buds and chemical pathways. In this study, both the taste and aroma obtained an almost similar pattern for sensory scoring, whereas the best accepted cookies were the ones from the control wheat flour without the addition of any gums. In contrast to the color scores, the cookies prepared with 6% cactus gum ranked the lowest for the taste and aroma. The lowering of the sensory scores for the taste and aroma with the presence of gums could be due to the bland taste of the added gums and also the entrapment of some aromatic compounds by the gums, which could not be released in the saliva and perceived by the sensory buds. 

Additionally, texture is thought to play a significant role in cookie palatability [34]. As with the flavor, the wheat-only cookies received the highest ratings for texture, whereas the wheat flour cookies made with 6% cactus gum received the lowest ratings. The sample’s poor sensory acceptability might also be confirmed by the texture instrument’s greatest hardness value. Additionally, the fact that the surface of the gum-filled cookies (Figure 3) is porous, cracked, and has a few comparatively dark patches could account for the panelists’ less favorable responses to the texture. 

In the case of overall acceptability, all the samples received a sufficiently positive and higher response from the panelists, with scores in the range of 6.5–8.25. However, the cookies baked from the dough with the gums were found to be less acceptable compared to the control cookies, except for the cookies with 3% acacia gum, which received a sensory acceptance which was somewhat similar to that of the control. Similarly, the cookies with the 3% cactus gums remained the next preferred sample. Nonetheless, all the cookies manufactured from the dough with 6% gum were found to be the least acceptable, with the lowest score of 6.5 for the cookies with 6% cactus gum. Thus, gum addition negatively impacted the overall quality and acceptability of the cookies; hence, 3% acacia gum was a suitable level for cookie manufacturing. In line with the current findings, Mohamed, Alamri, Hussain, Ibraheem, Qasem, Shamlan, and Ababtain [6] also found that the cookies prepared with wheat flour with the addition of ziziphus gum and cordia gum remained less acceptable than the cookies prepared using only wheat flour. Conversely, the addition of prickly pear peel powder in the cookies at 20% and 30% improved the overall acceptability score of the cookies compared to the cookies without the powder [37]. 

## 3. Materials and Methods

### 3.1. Collection and Preparation of Raw Materials

Wheat flour and baking aids were purchased from the local market. Lactic acid, sugar, and sodium bicarbonate were supplied by Sigma Aldrich (St. Louis, MO, USA). The acacia and cactus gums were extracted from the material collected from the local plants according to the methods explained by Hussain, Mohamed, Alamri, Ibraheem, Qasem, Alsulami, and Ababtain [15]. The acacia gum was labeled as AG, while the cactus gum was CG.

### 3.2. Preparation of Flour Gum Blends

The wheat flour and gum blends were prepared by replacing flour at the 3 and 6% levels with the AG and CG. The resultant blends were dry mixed, sieved, and stored in airtight jars for further analysis and baking.

### 3.3. Pasting Properties of Flour Gum Blends

The Rapid Visco-Analyzer was used to measure the flour–gum paste characteristics (Newport Scientific, Sydney, Australia). Flour samples (3.5 g at 14% moisture) were directly weighed into aluminum canisters and adjusted to 28 g with distilled water. The flour–water slurry was heated at 13.15 °C/min to 95 °C in 3.42 min and maintained at that temperature for 3.30 min. In 3.48 min (at 12.93 °C/min), it was cooled to 50 °C and maintained for 2 min. The paddle rotated at 960 rpm for 10 s, then 160 rpm for the rest of the experiment. Data were collected in triplicate and processed using Thermocline window software according to the method followed by Shahzad et al. [50].

### 3.4. Dough Mixing Properties

The micro-dough lab (Perten Instruments; Sydney, Australia) was used to find the optimum water absorption capacity, which peaked at 500 farinograph units (FU). The samples (4 g at 14% moisture) were stirred at a speed of 63 rpm and a temperature of 30 °C for 20 min. After a mixing curve was made, the dough’s development time (min), stability time (min), softening time (FU), mixing tolerance index (FU), and quality number were found according to the method followed by Dangi et al. [51]. 

### 3.5. Dough Extensibility Properties

Dough extensibility was measured according to the method followed by Tebben et al. [52], with slight modifications. The TA-XT Plus (Stable Micro Systems, Godalming, Surrey, UK) Texture Analyzer’s Kieffer dough and gluten extensibility apparatus was used to measure the uniaxial extensibility. A lubricated (paraffin oil) Teflon molder was used to press about 10 g of the prepared dough, which was then allowed to rest at room temperature for 45 min. A strip of dough was taken out of the molder and clamped between the plates of the Kieffer rig. We used a trigger force of 5 g and a test speed of 3.3 mm/s. The pre-test and post-test speeds were 2.0 mm/s and 10.0 mm/s, respectively. The peak force and distance at the peak force, respectively, were recorded as the resistance to extension (g) and extensibility (mm).

### 3.6. Solvent Retention Capacity of Flour Gum Blends

AACC method no. 56-11 was used to measure the solvent retention capacity of the flour gum blends [36,53]. As solvents, we used double-distilled water, 50% sugar, 5% sodium bicarbonate, and 5% lactic acid. Twenty-five milliliters of solvents was added to 2.0 g of flour in 30 mL tubes and centrifuged at 1000× *g* for 15 min (Herolab GmbH laboratory equipment Ludwig-Wagner-Str. 12 D-69168 Wiesloch, Germany). After decanting the liquid, the weight of the precipitated gels was recorded and the %SRC was determined using following expression.

$$SRC\;\left(\%\right)=\frac{wet\;pellet\left(g\right)}{\left[flour\;weight\left(g\right)-1\right]}\times \frac{86}{\left[100-flour\;moisture\;\left(\%\right)\right]}\times 100$$

### 3.7. Preparation of Cookies

The cookies were made with the control and blended flours using the method described by Hussain et al. [54]. The ingredients were precisely weighed. Shortening (30 g) and sugar (50 g) were mixed together, and eggs (1 no = 80g) were added. The flour blends (100 g) and baking powder (2 g) were sifted together and added to the sugar–shortening–egg mixture, which was then mixed to a homogeneous mass. The dough was then rolled with a roller (5 mm thickness), and the cookies were cut with a 30 mm-diameter cutter. The cookies were baked for 10 min in trays lined with butter paper at 200–210 °C. The baked cookies were allowed to cool for 30 min before being stored in airtight jars for further investigation.

### 3.8. Physical Evaluation of Cookies

The method described by Hussain, Anjum, Butt, Khan, and Asghar [54] was used to determine the cookie samples’ diameter (D), thickness (T), and spread factor (SF). By rotating the cookies at an angle of 90 degrees three times, the average diameter of six cookies arranged in a line was estimated using a Vernier caliper. The average cookie diameter was given in millimeters. In a similar manner, six cookies were stacked on top of one another, and the average cookie height was determined in millimeters by switching the placement of the cookies. The ratio of the diameter to the thickness was used to calculate the spread factor.

### 3.9. Texture Analysis of Cookies

The three-point bending rig on the heavy-duty platform and the 50 kg load cell of the TA-XT Plus instrument were utilized in the performance of the texture analysis of the cookie samples. The pre-test speed was 1 mm/s, the test speed was 3 mm/s, and the post-test speed was 10 mm/s. All of these speeds were used during the test. The information was recorded three times as a hardness in grams (maximum force that could be applied before breaking) and the fracturability in mm (the distance travelled by the probe before the breaking of the sample).

### 3.10. Color Parameters of the Cookies

The color values of the cookie samples, including L* (lightness), a* (redness), and b* (yellowness), were measured with a Minolta color grader that was equipped with a D65 light source according to the method followed by Alamri [55].

### 3.11. Sensory Evaluation of Cookies

Ten expert assessors, including postgraduate students and teachers from the Department of Food Science and Nutrition at King Saud University in Riyadh, Saudi Arabia, were involved in the sensory evaluation. The judges assessed the cookies’ sensory qualities, including taste, aroma, color, texture, and overall acceptability. The hedonic scale included nine points, with nine meaning “like very much”, five meaning “neither dislike nor like”, and one meaning “highly despise”. Cookies having a sensory attribute score of more than 5 or above the average were deemed to be acceptable [56].

### 3.12. Statistical Analysis

All measurements were performed in triplicate, and the data were statistically analyzed using the one-way ANOVA test. Using the PASW^®^ Statistics 18 software and Duncan’s multiple range test at *p* ≤ 0.05, the means were compared.

## 4. Conclusions

The addition of both gums at concentrations of 3 and 6% decreased the water absorption but slowed the dough formation. In the presence of cactus gum, the dough stability and quality were reduced, whereas the softening and mixing tolerance rose. The acacia and cactus gums improved the extensibility and the resistance to extension of the dough at both levels. The incorporation of acacia and cactus gums enhanced the texture of the cookies. The cookies with a lower level of gum were harder, while the cookies with cactus gum had a higher ratio of spread ability. Overall, the cookies containing gum were acceptable from a sensory standpoint. Locally grown cactus and acacia can be used to make cookies that have more soluble fiber than the control cookies.

## Figures and Tables

**Table 1:** Pasting properties of flour gum blends.

	PV (cP)	FV (cP)	Breakdown (cP)	SB (cP)	Peak time (min)	Pasting Temp (PT) (°C)
Control (100% WF)	1888 ± 43.10 ^b^	2056 ± 2.10 ^a^	734 ± 26.50 ^a^	902 ± 14.50 ^a^	5.83 ± 0.04 ^b^	69.40 ± 0.05 ^b^
3% Cactus gum	1846 ± 13.50 ^b^	1992 ± 16.23 ^b^	702 ± 2.10 ^b^	847 ± 0.50 ^b^	6.07 ± 0.01 ^a^	68.55 ± 0.03 ^c^
6% Cactus gum	1939 ± 8.10 ^a^	2028 ± 9.12 ^b^	749 ± 2.50 ^a^	838.5 ± 3.5 ^c^	6.10 ± 0.03 ^a^	68.90 ± 0.40 ^c^
3% Acacia gum	1453 ± 8.10 ^c^	1592 ± 31.50 ^c^	567 ± 11.50 ^c^	706 ± 12.2 ^d^	5.56 ± 0.04 ^c^	84.05 ± 0.80 ^a^
6% Acacia gum	1119 ± 16.20 ^d^	2028 ± 9.15 ^b^	445 ± 6.10 ^d^	588 ± 4.10 ^e^	5.51 ± 0.03 ^c^	84.90 ± 0.02 ^a^

WF = wheat flour; PV = peak viscosity; FV = final viscosity; SB = setback viscosity; cP = centipoise; PT = pasting temperature. Values followed by different letters (upper case) in columns are significantly different at *p* < 0.05.

**Table 2:** Effect of acacia and cactus gums on the dough mixing properties.

	WA (%)	DDT (min)	Stability (min)	Softening (FU)	MTI (FU)	Quality Number
Control (100% WF)	61.47 ± 0.36 ^a^	1.60 ± 0.08 ^e^	5.70 ± 0.22 ^b^	91.67 ± 2.36 ^d^	35.67 ± 4.19 ^e^	61.23 ± 0.95 ^a^
Cactus 3%	58.93 ± 0.09 ^b^	3.93 ± 0.09 ^d^	3.40 ± 0.08 ^c^	146.63 ± 1.73 ^b^	111.00 ± 2.94 ^b^	40.97 ± 1.19 ^d^
Cactus 6%	56.50 ± 0.24 ^c^	4.63 ± 0.05 ^c^	2.73 ± 0.05 ^d^	152.80 ± 2.36 ^a^	117.33 ± 2.05 ^a^	37.87 ± 0.61 ^e^
Acacia 3%	58.63 ± 0.12 ^b^	5.57 ± 0.26 ^b^	7.40 ± 0.16 ^a^	99.67 ± 0.47 ^c^	52.33 ± 2.05 ^d^	56.43 ± 0.39 ^b^
Acacia 6%	56.00 ± 0.16 ^d^	6.10 ± 0.15 ^a^	5.40 ± 0.22 ^b^	75.00 ± 4.08 ^e^	62.00 ± 0.78 ^c^	49.93 ± 1.28 ^c^

WF = wheat flour; WA = water absorption; DDT = dough development time; MTI = mixing tolerance index; FU = farinograph units. Values followed by different letters (upper case) in columns are significantly different at *p* < 0.05.

**Table 3:** Textural and physical properties of the cookies.

	Hardness (grams)	Fracturability (mm)	Thickness (mm)	Diameter (mm)	Spread Ratio
Control (100% WF)	2443.76 ± 83.35 ^d^	0.37 ± 0.02 ^e^	9.34 ± 0.10 ^b^	54.56 ± 0.16 ^c^	5.84 ± 0.05 ^d^
3% Cactus gum	2668.76 ± 83.35 ^c^	0.39 ± 0.02 ^d^	8.81 ± 0.09 ^c^	53.53 ± 0.10 ^d^	6.01 ± 0.06 ^b^
6% Cactus gum	5110.25 ± 126.47 ^a^	0.45 ± 0.01 ^c^	8.42 ± 0.01 ^d^	52.06 ± 0.34 ^e^	6.18 ± 0.05 ^a^
3% Acacia gum	2408.37 ± 76.74 ^d^	0.54 ± 0.02 ^b^	9.45 ± 0.10 ^b^	55.63 ± 0.23 ^b^	5.92 ± 0.08 ^c^
6% Acacia gum	3354.77 ± 99.48 ^b^	0.65 ± 0.05 ^a^	10.07 ± 0.04 ^a^	56.37 ± 0.26 ^a^	5.60 ± 0.02 ^e^

Diameter/thickness = spread ratio. Values followed by different letters in columns are significantly different at *p* < 0.05.

**Table 4:** Color parameter of the cookies.

	L*	a*	b*
Control (100% WF)	79.64 ± 0.22 ^b^	−0.72 ± 0.06 ^d^	28.06 ± 0.69 ^a^
3% Cactus gum	78.57 ± 1.15 ^c^	−2.37 ± 0.84 ^b^	27.59 ± 0.95 ^a^
6% Cactus gum	76.71 ± 0.57 ^d^	−2.91 ± 0.20 ^a^	25.51 ± 0.23 ^b^
3% Acacia gum	80.43 ± 0.62 ^a^	−2.46 ± 0.55 ^b^	25.48 ± 0.80 ^b^
6% Acacia gum	78.61 ± 0.14 ^c^	−1.80 ± 0.18 ^c^	25.78 ± 0.56 ^b^

L* = lightness; a* = green/red; b* = blue/yellow. Values followed by different letters in columns are significantly different at *p* < 0.05. Values followed by different letters in columns are significantly different at *p* < 0.05.

## Data Availability

Not applicable.
